# Plurihormonal pituitary macroadenoma:  a case report

**DOI:** 10.1186/s13256-021-02948-6

**Published:** 2021-07-29

**Authors:** Ebtesam Allehaibi, Mussa H. AlMalki, Imad Brema

**Affiliations:** 1 Obesity, Endocrine, and Metabolism Center, King Fahad Medical City, Riyadh, Saudi Arabia; 2 King Fahad Medical City, Faculty of Medicine, King Saud bin Abdul-Aziz University for Health Sciences, Riyadh, Saudi Arabia

**Keywords:** Plurihormonal pituitary adenoma, Nonfunctioning, Silent pituitary adenomas, Silent somatotroph adenomas, Silent gonadotroph, Silent corticotroph

## Abstract

**Background:**

Plurihormonal pituitary adenomas are a unique type of pituitary adenomas that secrete two or more pituitary hormones normally associated with separate cell types that have different immunocytochemical and ultrastructural features. Although they represent 10–15% of all pituitary tumors, only a small fraction of plurihormonal pituitary adenomas clinically secrete multiple hormones. The most common hormone combinations secreted by plurihormonal pituitary adenomas are growth hormone, prolactin, and one or more glycoprotein hormones. The most common hormonal symptom is acromegaly (50%). The aim of this case report is to bring awareness about this rare type of pituitary adenomas and to describe the unique presentation of our patient, even though plurihormonal pituitary adenomas are known mostly as a clinically silent tumors.

**Case presentation:**

Herein, we describe an unusual case of plurihormonal pituitary adenoma with triple-positive staining for adrenocorticotropic hormone, growth hormone, and prolactin. The patient is a 65-year-old Egyptian woman who presented with mass effect symptoms of the pituitary tumor, which primarily manifested as severe headache and visual field defects. She also presented with some cushingoid features, and further analysis confirmed Cushing’s disease; slightly high prolactin and normal growth hormone levels were observed. She underwent transsphenoidal surgery and has been in remission thus far. Only a few cases have been reported in the literature, but none has exhibited silent acromegaly or mass effect symptoms as the initial presentation.

**Conclusion:**

This case highlights an unusual plurihormonal pituitary adenoma case with a rare combination of secreted hormones; mass effect symptoms were dominant, as were uncommon visual field defects. Our case further proves that immunohistochemical analyses of all pituitary hormones are needed to ensure correct diagnosis and to alert clinicians to the need for more rigorous follow-up due to the higher morbidity of these patients. Our case report approval number Federal Wide Assurance NIH, USA is FWA00018774 IRB registration number with OHRP/NIH is IRB00010471.

## Background

Plurihormonal pituitary adenomas (PHAs) are a unique type of pituitary adenoma that are further divided into two main groups: monomorphous adenoma of one cell type producing different hormones or bi- or plurimorphous adenoma of two or more cell types producing different hormones [1]. They represent 10–15% of all pituitary tumors, but only a small number clinically secretes multiple hormones. PHAs could be either functioning tumors, having symptoms related to one or more hormones secreted by the adenomas, or nonfunctioning tumors [2–4].

The most common hormone combinations secreted by PHAs are growth hormone (GH), prolactin (PRL), and one or more glycoprotein hormones. The most common hormonal symptom is acromegaly (50%), followed by subclinical Cushing’s disease, then symptoms related to hyperprolactinemia [5].

Careful follow-up of patients with PHA is essential owing to the high risk of tumor recurrence as compared with tumors that secrete only one hormone [1, 6]. This report describes an unusual case of pituitary adenoma presenting with features of Cushing’s disease, and the resected tumor revealed triple hormonal staining for ACTH, GH, and prolactin.

## Case presentation

A 65-year-old Egyptian woman with a history of type 2 diabetes, hypertension, primary hypothyroidism, and class-III obesity presented acutely to the emergency room (ER) with an intractable headache and blurred vision. She had also recently experienced a worsening of her diabetes control despite being on a basal-bolus insulin regimen. She had no clear-cut cushingoid features apart from central obesity, and she had no family history of pituitary tumors or similar illness. She is a stay-at-home mother to four. Children are all a product of uneventful pregnancies and were delivered normally. She is married for 20 years to a physician. She never smoked or drank alcohol.

**Fig. 1:**
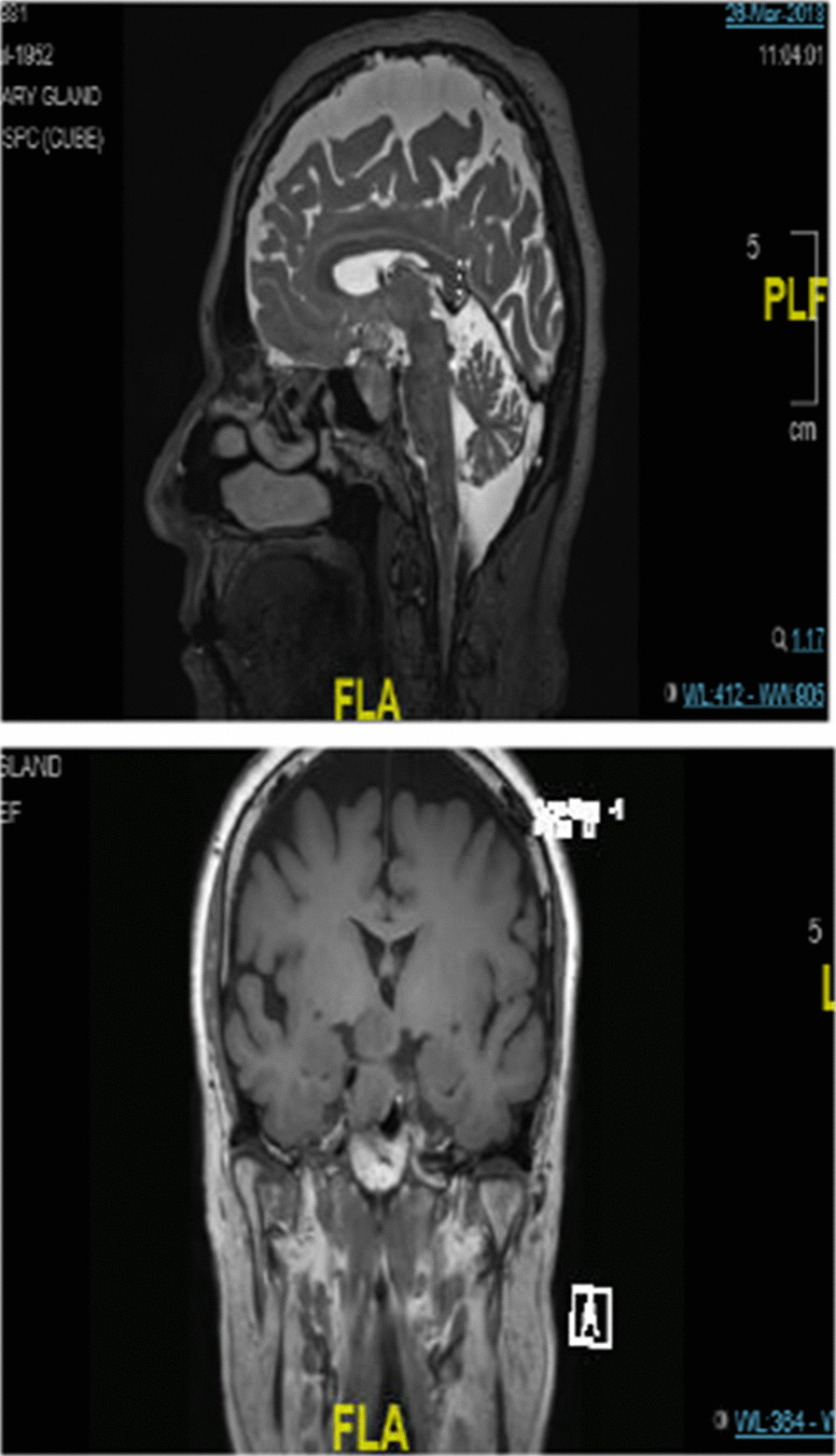
Baseline magnetic resonance imaging (MRI) pituitary showing a large sellar/suprasellar mass lesion causing compression and superior displacement on the optic chiasm as well as compression and lateral displacement of the neurohypophysis. Erosion of the roof of the sphenoid sinus with subsequent extension is also observed

**Fig. 2:**
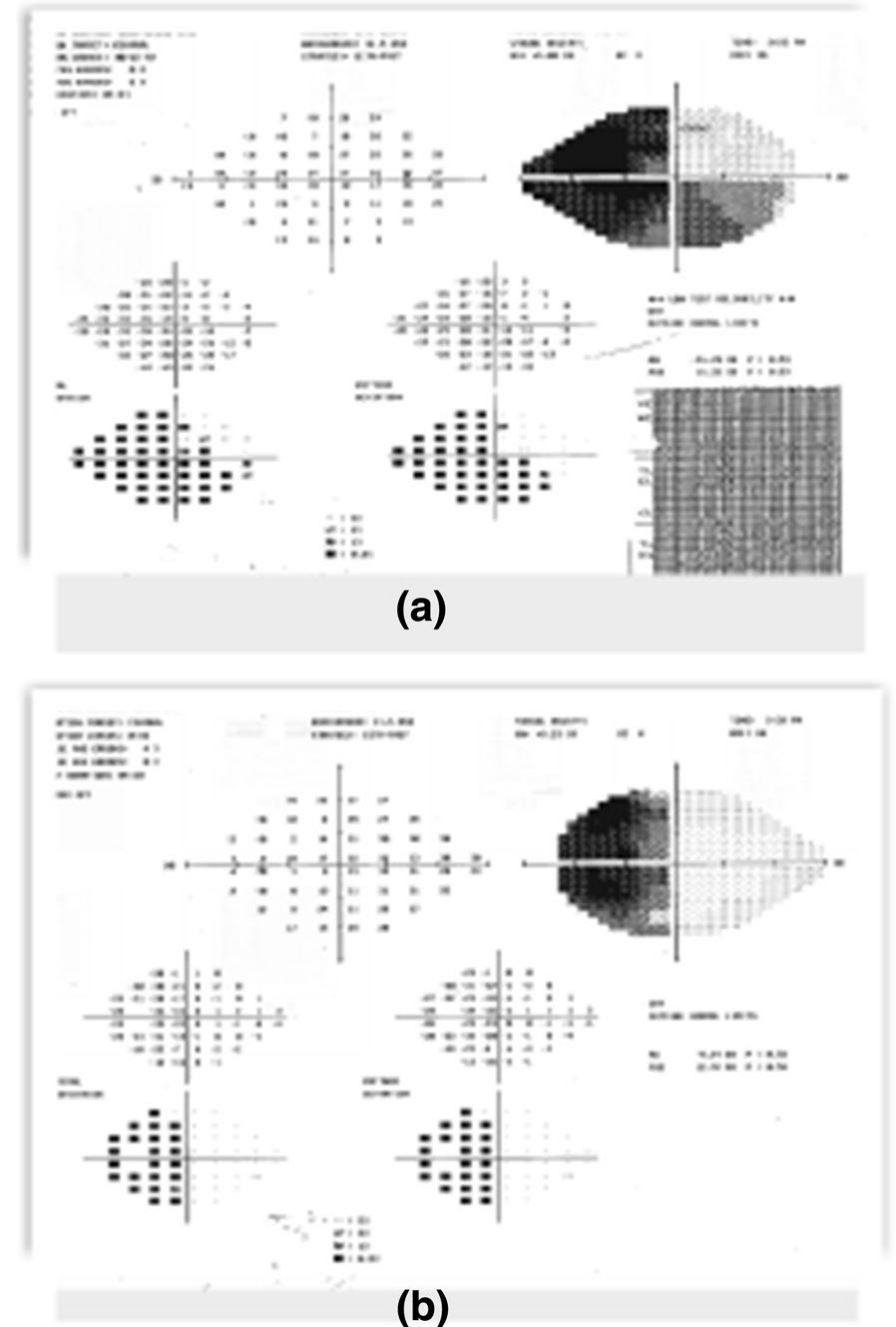
**a** Preoperative visual field examination.** b** Post operative visual field examination; the patient had left homonymous hemianopia and unilateral right inferior quadrantanopia

**Fig. 3:**
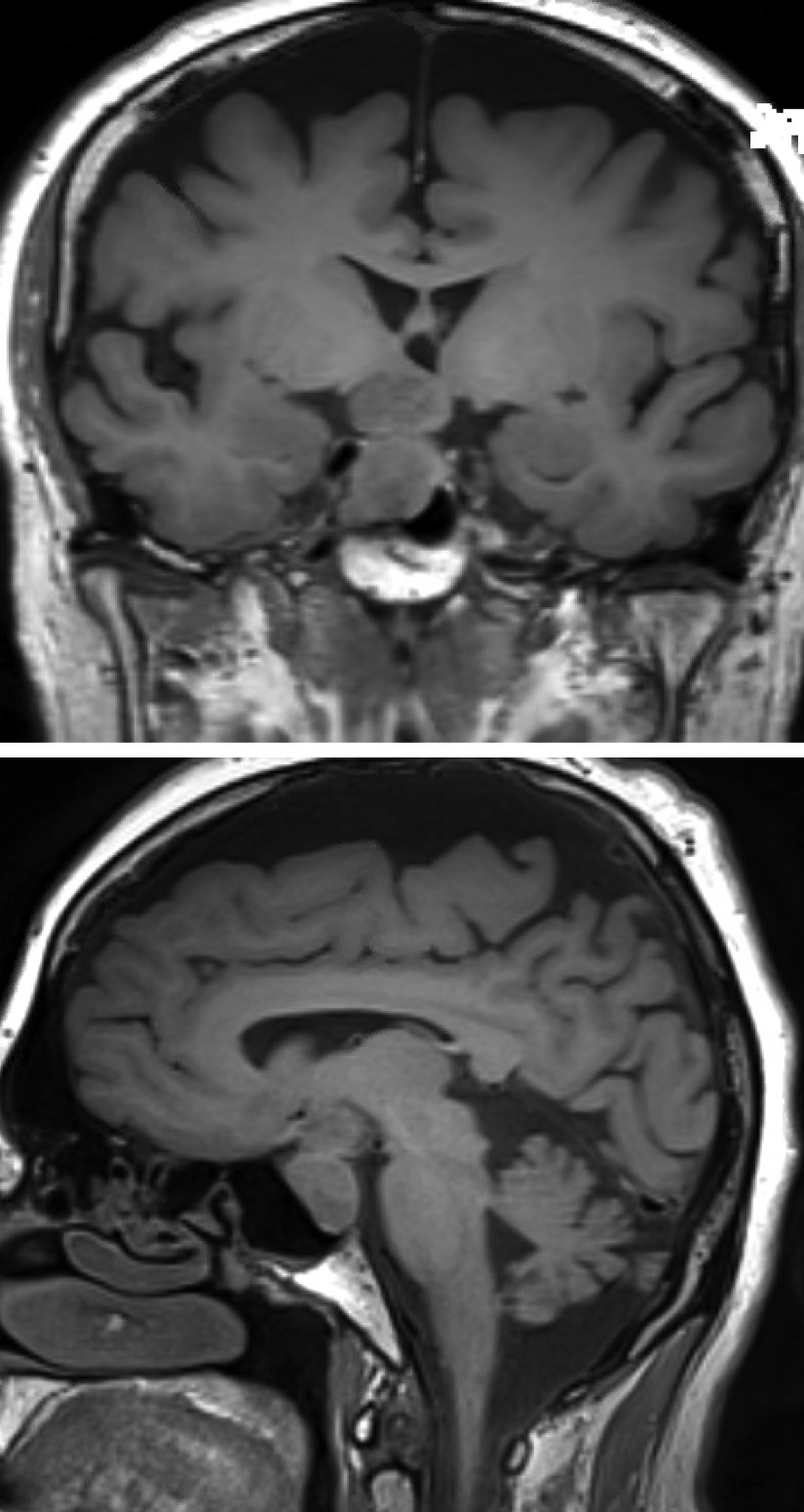
Postoperative pituitary MRI revealing residual isointense enhancement of the tumor in the sella and along the stalk measuring 9.9 × 9.9 mm in the anteroposterior (AP) and transverse dimensions and 1.3 cm in the craniocaudal (CC) dimension. The tumor is closely adherent to the posterior aspect of the optic chiasm

**Fig 4:**
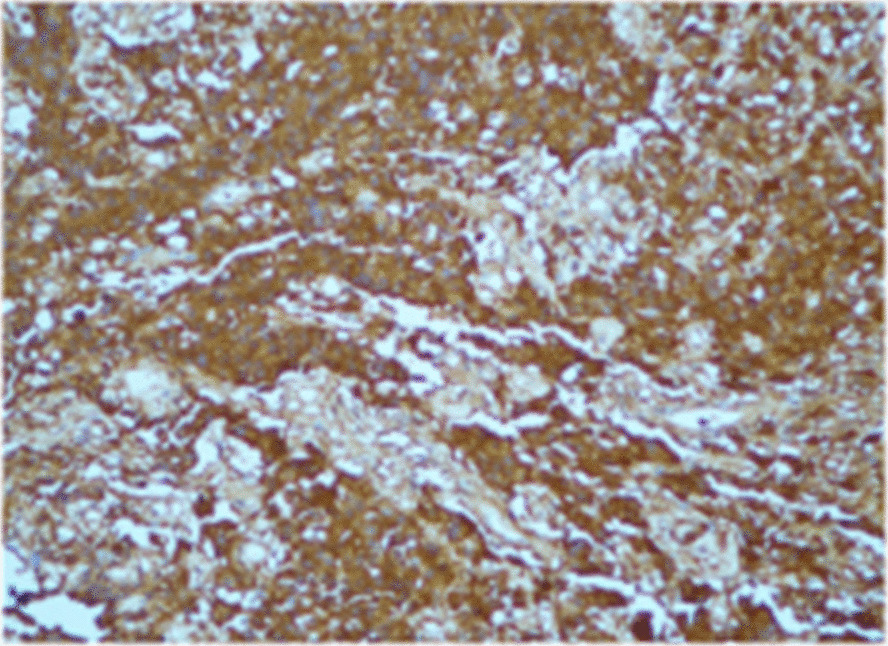
Immunostaining showing positivity for adrenocorticotropic hormone

**Fig. 5:**
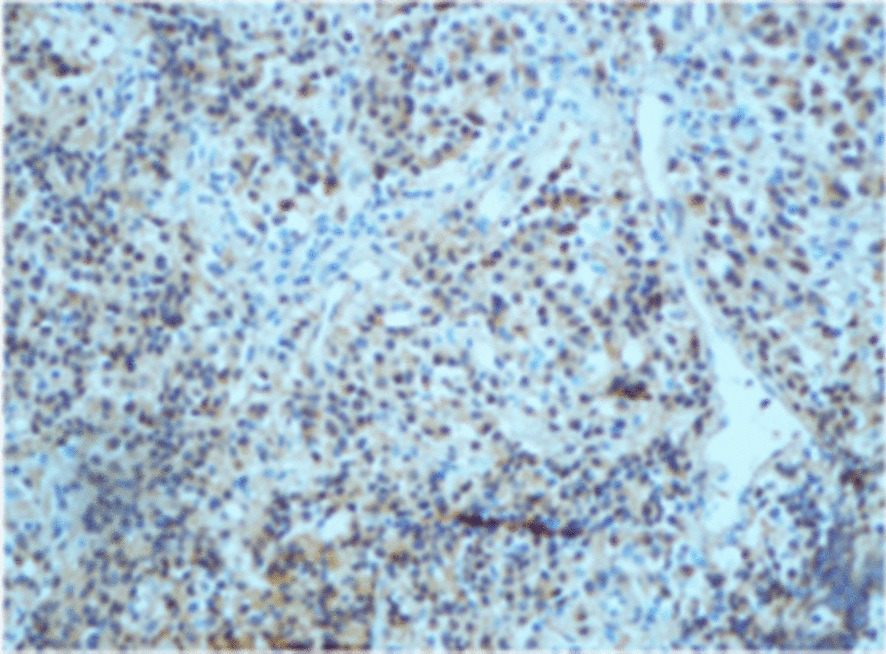
Immunostaining showing positivity for growth hormone

**Fig. 6:**
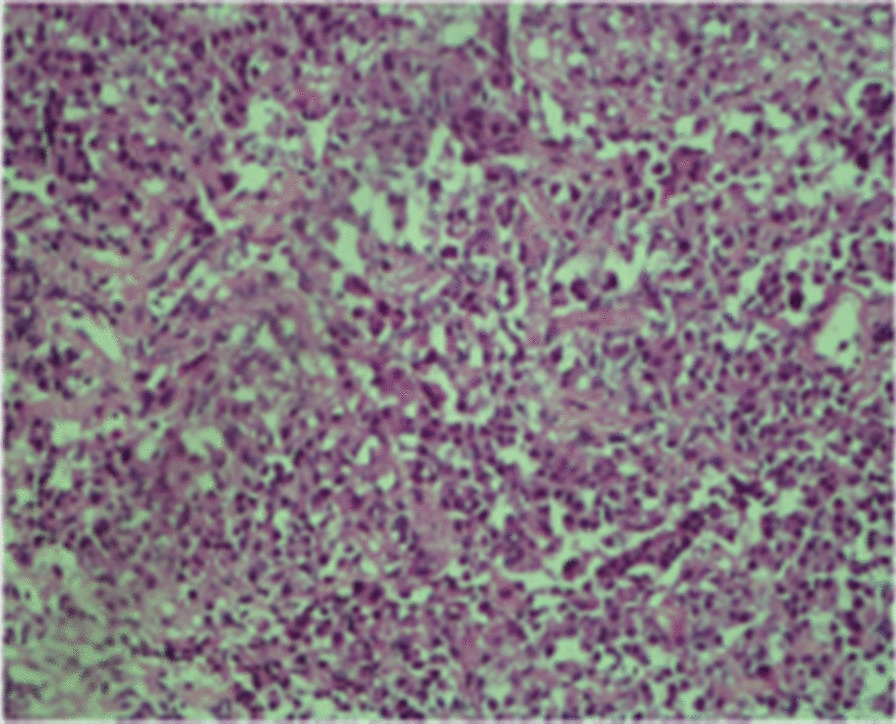
Immunostaining showing positivity for prolactin

**Fig. 7:**
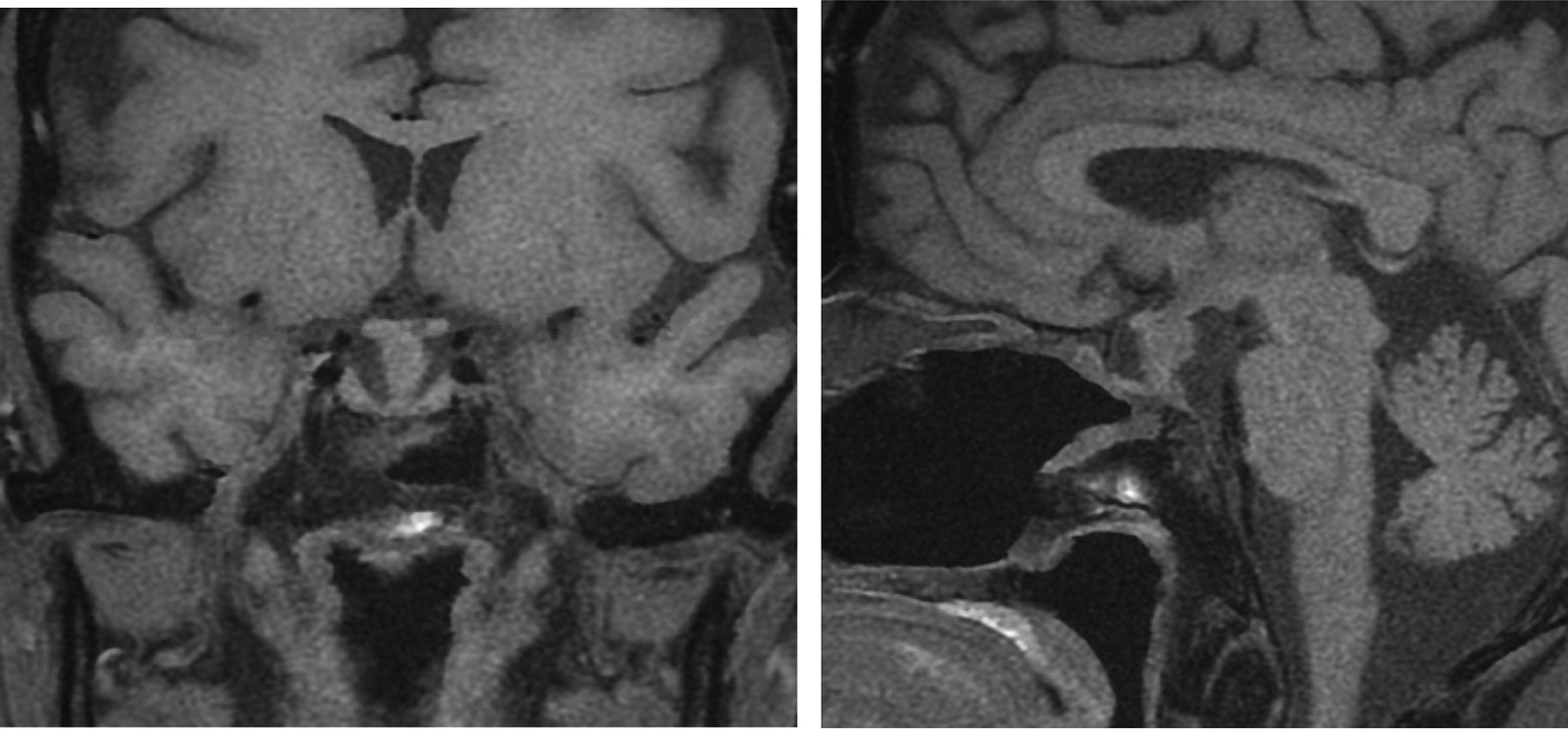
Last pituitary magnetic resonance imaging (MRI) 15/10/2019. Compared with the previous MRI dated on 15 April 2019, there is redemonstration of the residual enhancing lesion seen in the suprasellar region closely related to the pituitary stalk. Its residual remained stable in size; measures 12.5 × 11.5 mm in CC × AP dimensions in sagittal plane compared with 11.5 × 11.5 mm in previous study in same location. No other significant interval changes demonstrated

Computed tomography (CT) brain imaging and subsequent magnetic resonance imaging (MRI) showed a sellar mass consisting of a pituitary macroadenoma that measured 2.9 × 1.5 cm invading the roof of the sphenoid sinuses and compressing the neurophysis and the optic chiasm (Fig. 1). She has central obesity but did not appear cushingoid or acromegalic. Visual field examination revealed left homonymous hemianopia and right homonymous inferior quadrantanopia (Fig. 2A). Initial laboratory investigations revealed elevated corticotropin (ACTH) levels of 33.6 (1.03–10.7) pmol/L, mildly increased prolactin levels of 66.3 (5.18–26.53) ng/ml , normal IGF-1 levels of 17.29 (4.68–31.72) nmol/L, and normal GH levels of 0.72 (0.18–20.6) mIU/L. The remaining anterior pituitary hormone levels were as follows: FSH was 12.50 (4.5–21.5) IU/L, LH was 3.5 (9–19) pmol/L, TSH was 1.441 (2.6–5.7) mIU/L, and free T4 was 13.1 IU/L (9–23). Further investigations confirmed Cushing’s disease as follows: 24-hour urinary free cortisol was grossly elevated at 391 µg/24 hours (6–123), serum cortisol after the 1 mg dexamethasone suppression test was 783 nmol/l (normal < 50 nmol/l). Serum cortisol after the high-dose dexamethasone suppression test was 613 nmol/l (20% reduction from baseline). She underwent uneventful transsphenoidal surgery (TSS) for decompression of the optic chiasm, which was successful at normalizing her visual fields; however, residual tumor was still present on follow-up MRI after 3 months (Fig. 3). Interestingly, histology of the resected tissue showed staining for ACTH (Fig. 4), GH (Fig. 5), and PRL (Fig. 6), with a Ki-67 proliferation index of less than 2%. Postoperatively, she experienced symptom resolution with normalization of the visual fields (Fig. 2B). A follow-up evaluation 12 months postoperatively showed a normal response to the 1 mg dexamethasone suppression test; her cortisol levels were < 27.6 nmol/L, and 24-hour urinary free cortisol was also normal at 177 µg/24 hours (21–292). MRI (Fig. 7) pituitary performed 17 months postoperatively showed redemonstration of the residual enhancing lesion seen in the suprasellar region closely related to the pituitary stalk, which remained stable in size, measuring 12.5 × 11.5 mm. Her diabetes mellitus and hypertension also became controlled on fewer medications with ability to discontinue insulin therapy. She remained asymptomatic with no biochemical evidence of recurrence 17 months postoperatively, and there was no need for any treatment utilization.

## Discussion

The majority of plurihormonal pituitary adenomas produce GH, PRL, and TSH because somatotroph, lactotroph, and thyrotroph cells all arise from the same progenitor [7]. The expression of pituitary hormones is regulated by several transcription factors: PIT-1 regulates the functional differentiation of GH, PRL, and TSH; STF-1 and GATA-2 regulate the expression of FSH and LH, while ACTH expression is controlled by T-PIT [7, 8], which might explain the higher association with GH and PRL cosecretion. PHAs constitute a significant proportion of pituitary adenomas, with a prevalence of approximately 31–36% of surgically resected tumors [9, 10]. The most frequent associations are with GH and PRL or LH and FSH [11]. GH-producing adenomas with concomitant ACTH production are extremely rare, although they have been reported previously in a few cases [12–20]. Clinically, the majority of PHAs are silent, and diagnosis almost always relies on immunohistochemical analysis of the tumor tissue to demonstrate positivity for unrelated hormones [8, 9]. Roca *et al.* [20] recently reviewed the literature on PHAs and reported 21 cases with ACTH–GH plurihormonal pituitary adenoma. Of these, 2 cases had Cushing’s disease, 5 had both acromegaly and Cushing’s disease, 11 had signs of acromegaly, and 3 had pituitary apoplexy. Interestingly, they found that six cases had PRL secretion in addition to ACTH and GH. In PHAs, symptoms related to ACTH are uncommon and were observed in only 3.6% of the reported cases; this phenomenon may be due to insufficient autonomous ACTH production or the absence of ACTH production [21–23]. The pathogenesis of plurimorphous plurihormonal tumors is less clearly defined, but it has been suggested that it may result from neoplastic transformation of two separate cell lines or from the transdifferentiation of one single tumor cell line into a different hormone-producing cell line [8].

In the current study, we present an interesting and rare case of PHA in which the patient had ACTH-producing tumors that clinically manifested as Cushing’s disease and showed GH and PRL positivity by immunohistochemistry, both of which were asymptomatic or silent. In contrast to previously published cases where there was a predominant clinical presentation with acromegaly and symptoms of hyperprolactinemia, our patient presented initially with headaches and visual symptoms, as well as with clinical features of Cushing’s syndrome in the form of uncontrolled blood pressure, diabetes, obesity, and moon face. Postoperative pituitary MRI scans showed a successful reduction in tumor size with some residual tumor remaining in the sella, leading to the resolution of the biochemical and clinical features of cortisol excess. These rare and unusual PHAs tend to be aggressive and associated with a poor outcome [15]. PHA patients with ACTH cosecretion appear to have a higher rate of tumor recurrence. Careful evaluation of patients with such tumors and strict follow-up regimens are needed owing to the higher morbidity of these patients [15, 24, 25]. Our case adds a significant body of knowledge to the current literature of such rare tumors. However, further studies are needed to elucidate the features and natural history of these PHAs.

## Conclusion

This case highlights an unusual PHA case of a rare pituitary adenoma with cosecretion of ACTH, GH, and PRL. Mass effect symptoms were dominant, along with some cushingoid features. Although PHAs are usually clinically silent tumors, they are not uncommon pituitary adenomas, and immunohistochemical staining for all pituitary hormones is needed to ensure the correct diagnosis and to alert the clinicians to the need for more rigorous follow-up due to the higher morbidity of these patients. Further work is required to better understand the pathogenesis of this rare condition.

## Data Availability

The datasets used and/or analyzed during the current study are available from the corresponding author upon reasonable request.

## Abbreviations

GH: Growth hormone
PRL: Prolactin
ACTH: Adrenocorticotropic hormone
PTHs: Plurihormonal pituitary adenomas
FSH: Follicle-stimulating hormone
TSS: Transsphenoidal surgery
